# Personalized Superficial HDR Brachytherapy—Dosimetric Verification of Dose Distribution with Lead Shielding of Critical Organs in the Head and Neck Region

**DOI:** 10.3390/jpm12091432

**Published:** 2022-08-31

**Authors:** Grzegorz Zwierzchowski, Grzegorz Bielęda, Agata Szymbor, Marek Boehlke

**Affiliations:** 1 Electroradiology Department, Poznan University of Medical Sciences, 61-866 Poznań, Poland; 2 Medical Physics Department, Greater Poland Cancer Centre, 61-866 Poznań, Poland; 3 Faculty of Physics, Adam Mickiewicz University in Poznań, 61-614 Poznań, Poland; 4 Medical Physics Department, West Pomeranian Oncology Center, 71-730 Szczecin, Poland

**Keywords:** brachytherapy, dosimetry, dose calculations, quality assurance, 3D printing

## Abstract

Background: Surface brachytherapy, usually characterized by a high dose gradient, allows the dose to be precisely deposited in the irradiated area while protecting critical organs. When the lesion is located in the nasal or ocular region, the organ of vision must be protected. The aim of this study was to verify the dose distributions near critical organs in the head and neck region during a brachytherapy procedure using lead shielding of the eye. Methods: Anthropomorphic phantom using 3D-printing technology was prepared. The doses deposited at a point in the lens of the eye and on the surface of the eyelid, directly under the lead shield were calculated and measured using EBT3 radiochromic films. Comparison of doses planned in the treatment planning system using the TG-43 formalism, TG-186 formalism, and measured were also performed. Results: Comparing the planned and calculated doses with TG186 formalism it can be assumed that the use of lead shields is a method for protecting the organ of vision from the adverse effects of ionizing radiation. Conclusions: The decision to use a lead shield during facial surface brachytherapy procedures should be made on a patient-by-patient basis and based on model-based calculation methods recommended by TG186.

## 1. Introduction

Surface brachytherapy of the head and neck region is carried out mainly for the treatment of skin cancers. According to WHO estimates, 2–3 million non-melanoma skin cancers (NMSCs) are currently diagnosed worldwide per year, with one third of these cases being skin cancer. These figures are probably underestimated. In Europe, 40–130 cases of basal cell carcinoma (BCC) are diagnosed in 100,000 people, while 8–30 suffer from squamous cell carcinoma (SCC). BCC accounts for 80% of diagnosed skin cancers. It is characterized by slow growth and local malignancy, unlike SCC, which is much more likely to metastasize and is characterized by rapid growth and infiltration of neighboring tissues. Development factors of skin cancer include mainly overexposure to ultraviolet radiation, but also X-rays or HPV viruses. The risk of skin cancer increases with age, with the most common diagnosis of the disease occurring after the age of 80. Many of the patients receiving a skin cancer diagnosis are older patients, with other health problems that often disqualify them from surgical treatment under general anesthesia. A risk concerning superficial skin cancer lesions may be the infiltration of the lesion into structures located deeper or adjacent to the tumor, such as muscle or bone. This is especially true for lesions located in the head and neck region, where about 80% of all skin cancer cases are localized. Often, the lesions also have a significant impact on patients’ quality of life, constituting a serious cosmetic defect.

Brachytherapy is often the therapy of choice when surgical treatment or teleradiotherapy does not show superior efficacy. It is also recommended when the lesion is located in anatomical curvatures, such as the curvature of the nose and the orbital region. HDR (High Dose Rate) method allows dose deposition over a short time, in 2–3 times a week fractionation, which reduces the need for patient visits to the medical facility. Optimized dose distribution allows good coverage of the irradiated area with the prescribed dose while protecting adjacent structures. Brachytherapy is often used for Kaposi’s sarcoma, lymphoma, or keloid (scar tissue). Brachytherapy cannot be used to treat malignant melanoma, due to radiation resistance, skin cancers that infiltrate bone and cartilage or auricle, infiltrating the ear canal, and cancers of the upper eyelid [1].

Either superficial or interstitial brachytherapy is used to treat skin cancers, depending on the area of the lesion’s location. Leipzig™ and Freiburg Flap™ surface applicators are often used for more extensive lesions. Personalized applicators created individually for the patient, based on the CT imaging, and prepared using 3D printers, are also used [2]. If the size of the tumor changes, the applicator can be easily re-printed. Individual applicators can be created from suitable polymers, such as PLA (Polylactide), thermoplastic materials, or acrylic resin. These molds precisely adhere to the surface of the patient’s skin, and inside them, and guide tubes for the radioactive source are prepared. The path of a radioactive source in the catheters is spaced about 5 mm from the skin surface, which prevents the delivery of high doses to the skin surface. The use of surface applicators allows also for accurate coverage of irregular lesions on flat surfaces [2,3].

Many organs in the head and neck region need to be protected during teleradiotherapy and brachytherapy treatments. Surface brachytherapy, thanks to its high dose gradient, allows the appropriate dose to be deposited in the irradiated area while protecting critical organs. However, when the lesion is located in the nasal or ocular region, the organ of vision must be protected. Particularly the most vulnerable to radiation complications (with this technique) will be the lens of the eye, if the threshold dose is exceeded, cataracts can develop. A cataract is an opacification of the lens of the eye, leading to progressive deterioration of vision and eventually loss of vision. The time between the organ’s exposure to the dose and the appearance of lens opacity is called the latency period. Depending on the dose, the latency period can be as long as 8 years [4,5].

Ionizing radiation also has harmful effects on the cornea and retina. If the total dose of about 50 Gy (fractionated conventionally) is exceeded, radiation ulceration of the cornea may occur. During irradiation, the endothelium of the retinal capillaries may be damaged. Exceeding a fractional dose of more than 1.9 Gy can lead to the development of retinopathy and loss of visual acuity. The latency period for these effects ranges from four months to two and a half years. Due to the radiosensitivity of these organs, there is a need for lead shielding. There is also information in the literature about copper shields attached to contact lenses [6].

Recent literature lacks mentioning of dose distribution measurements during the use of lead eye shields during superficial brachytherapy. Most treatment planning systems currently use the AAPM TG-43 formalism (Task Group 43 of the American Association of Physicists in Medicine), which does not take into account the scattering effects associated with different material densities. However, the differences between the planned dose distributions and the Monte Carlo algorithm calculations are rather small for iridium 192Ir sources. The potentially important air gap between the Freiburg™ flap-type applicator and the skin surface has no significant effect on the dose distribution. In contrast, Lepizig™ and Valencia™ type applicators are equipped with a plastic cap that prevents scattered electrons from reaching the skin surface [7].

### Aim of the Study

The main aim of this study was to verify the dose distributions near critical organs in the head and neck region during brachytherapy procedure using lead shielding of the eye region.

Specific objectives included:

(1) Development of a fabrication procedure for an anthropomorphic phantom using 3D-printing technology.
(2) Development of a method to measure the dose deposited at a point in the lens of the eye and at a point on the surface of the eyelid directly under the lead shield, using EBT3 radiochromic film placed in the phantom.
(3) Comparison of the dose distribution calculated in the treatment planning system with the doses measured using the radiochromic films.
(4) Comparison of doses planned in the treatment planning system using the TG-43 formalism, which assumes full scattering conditions in water, and the TG-186 formalism—which takes into account the electron densities of the surrounding tissues and the geometry of the irradiated region.

## 2. Materials and Methods

**Figure 1:**
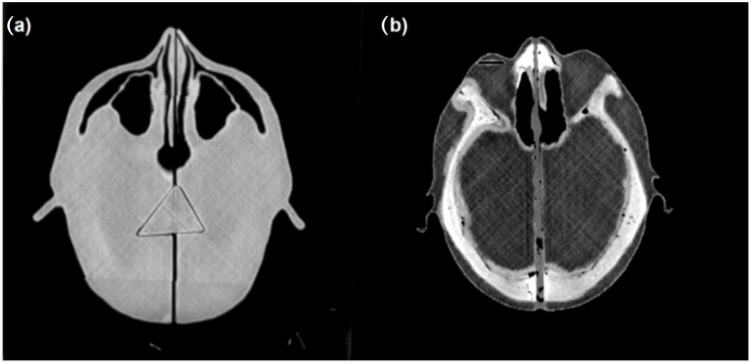
CT images of the phantom before (**a**) and after (**b**) filling of the bone spaces with plaster.

**Figure 2:**
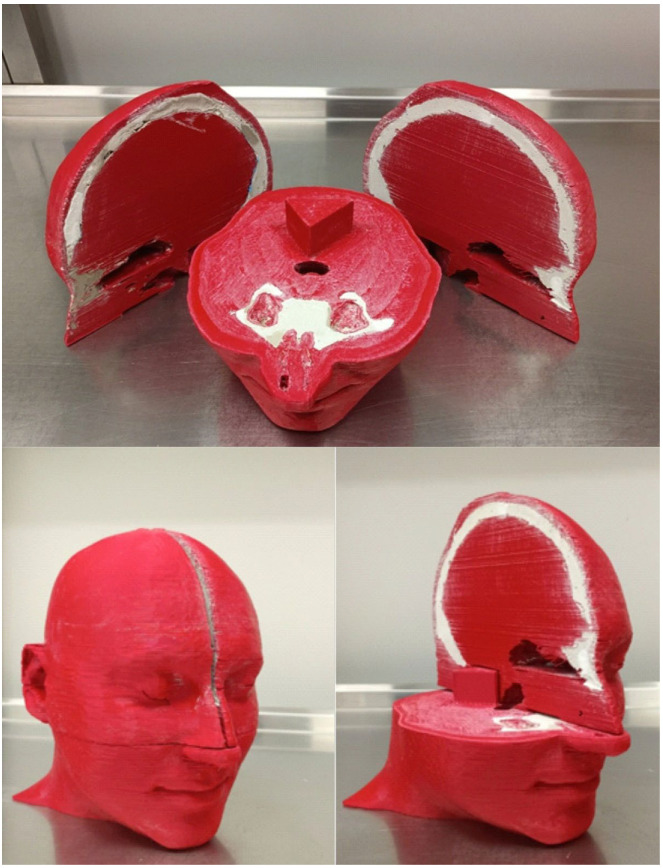
Finished 3D printed anthropomorphic head phantom used for purpose of this study.

For designing the anthropomorphic head phantom, the Beben^TM^ software was used. It was necessary for generating files according to STL (Standard Triangulation Language) format from DICOM (Digital Imaging and Communications in Medicine) files. The geometric data for the phantom project were prepared using the OncentraBrachy^TM^ v4.6 treatment planning system (Nucletron^TM^, Elekta^TM^) using CT data of the anonymized patient’s head. The cross-sectional layers of the CT scans were used to determine the volumes of the bones, eyes, lenses, and air spaces in the head and neck region. The created structures were then exported as an RTStructure file to Beben^TM^ software. Individual anatomical structures were generated as three-dimensional objects. The solids corresponding to the bone and air spaces were then subtracted from the volume of total head volume (external contour). In addition, a slit was placed in the right eye with a depth of 2.5 cm and measuring 1 cm × 0.2 cm. It was prepared to place the radiochromic film in it at the location corresponding to the lens of the eye. The phantom was divided into three parts to streamline the printing process. The volumes corresponding to the bones were filled with plaster. This material has a density close to the bone and a corresponding Hounsfield value of 600–700 HU. The use of plaster allowed a good reproduction of bone density and distinguished significantly from PLA with a density similar to soft tissue as seen in Figure 1. The finished phantom is presented in Figure 2.

**Figure 3:**
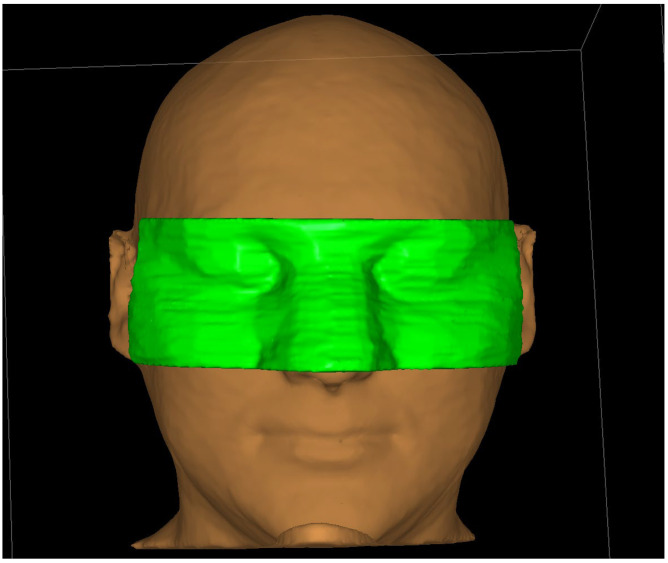
Personalized surface applicator for superficial brachytherapy (prepared as a bolus structure) which was used for purpose of this study.

A custom-printed personalized surface applicator was used to perform the phantom simulated treatment. The design of the custom applicator was based on CT images of the head phantom taken with 1 mm layers separation. The resulting images were imported into the OncentraBrachy^TM^ treatment planning system. The first step was to determine the outer contour of the phantom, using the lung window (width of 1600 HU with center at −600 HU). Using such settings allows to prepare an applicator that fits well on the facial surface. Using the transverse CT images, 10 sample target areas (CTV 1–10) were delineated on the facial surface, which were located at different linear and angular distances from the right eye. A bolus of 1 cm was then added to the outer contour of the patient at eye level. The added volume covers the area where the target volumes were located. The prepared bolus on the surface of the phantom outline is shown in Figure 3. At the height of the right eye, a space 2 cm wide and 0.5 cm deep was prepared to allow the lead shield to be inserted into the applicator.

**Figure 4:**
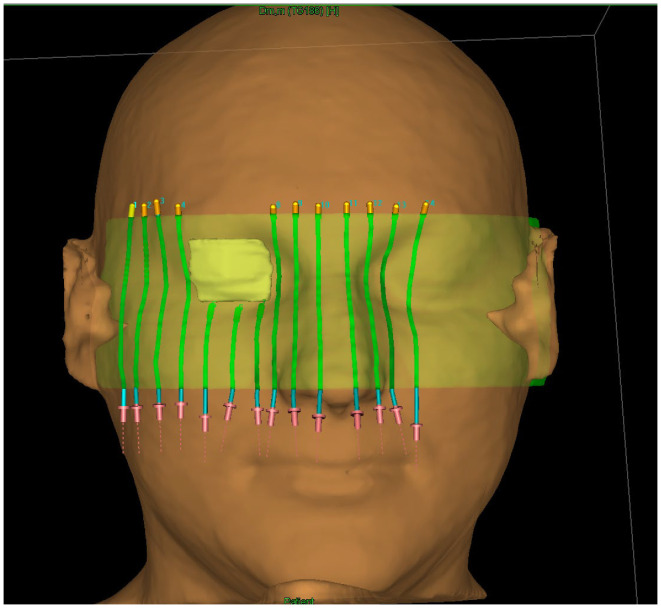
The applicator used for this study, with channels for brachytherapy catheters and space for the lead shield.

Using the treatment planning system, 14 channels for the source, spaced 0.5 cm from the skin surface and 1 cm from each other were designed. To create the channels in the applicator, a structure representing the volume of the applicator itself (RTStructure) was first exported to the Beben^TM^ program. Using the exported RTPlan file, the catheter paths inside the applicator were generated. The RTPlan file contains the coordinates of the source’s active stop positions in the same coordinate system as the coordinates of the applicator vertices. To ensure collision-free source insertion, the diameter of the channels was set at 3 mm. The applicator with channels for catheters and with space for the lead shield is shown in Figure 4.

**Figure 5:**
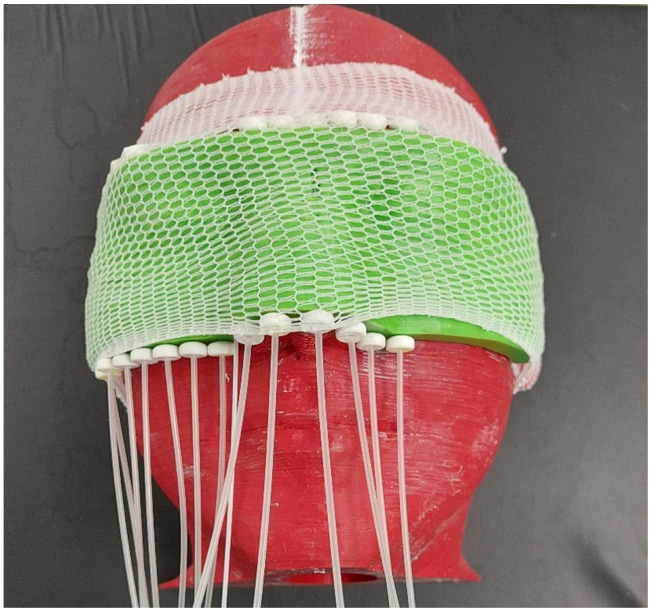
Head phantom with personalized brachytherapy applicator ready for CT imaging.

In order to finally prepare the treatment plan, the head phantom along with the 3D applicator fixed (Figure 5.) was imaged using a CT. The volumes of the critical organs and the target volumes were determined on the obtained images.

Using image fusion, the outlines of the eyes, lenses, and bones of the real patient were superimposed on the CT images of the phantom. In this way, it was possible to accurately delineate the critical organs in the CT cross sections of the printed phantom. Then the source travel paths in all channels of the designed applicator were reconstructed in the treatment planning system.

**Figure 6:**
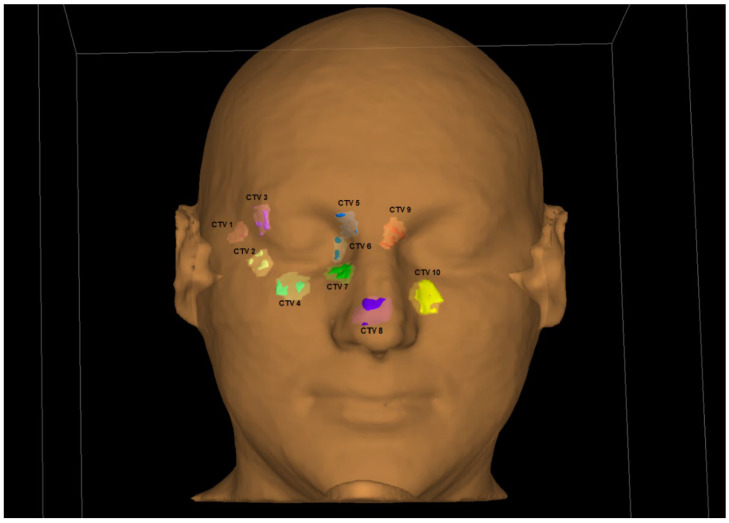
The location of the target areas (CTV1-CTV10) used for preparing the treatment plans.

For the purpose of this study 10 target areas located at different distances from the right eye were determined. They were located: on the right temple (CTV 1), in the outer corner of the right eye (CTV 2, CTV 3), under the eye (CTV 4), in the inner corner of the right eye (CTV 5, CTV 6, CTV 7), on the tip of the nose (CTV 8), and near the left eye (CTV 9, CTV 10). Subsequently, 10 treatment plans were created at TPS, for irradiating the corresponding CTV. The treatment plans were carried out in order from 1 to 10. The location of the target areas is shown in Figure 6.

**Figure 7:**
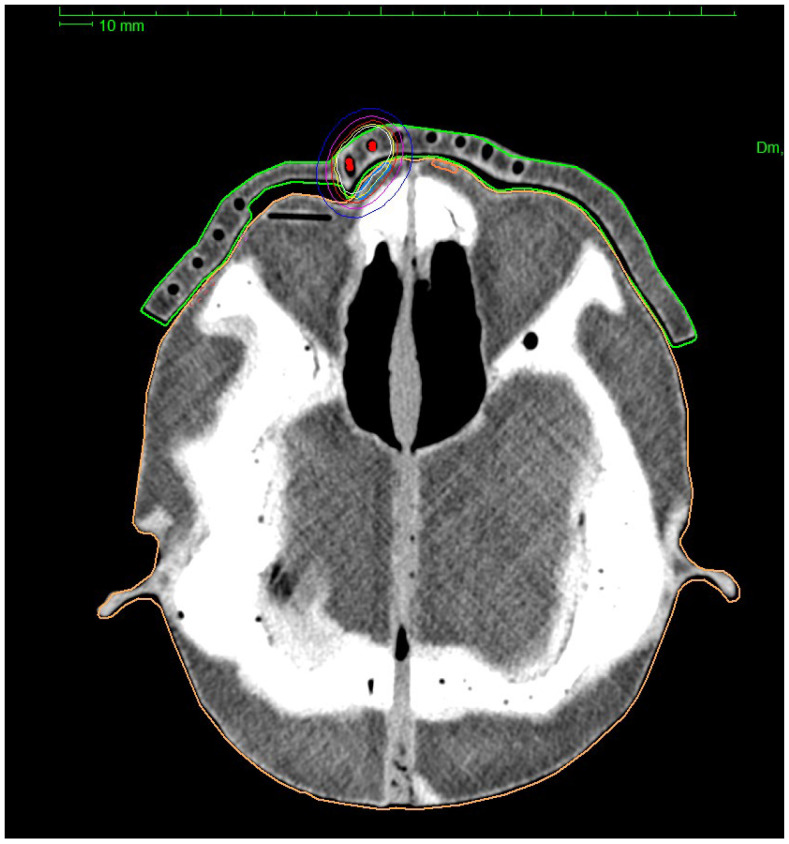
Sample dose distribution obtained during the treatment planning process with clearly visible channels for brachytherapy catheters inside a 3D printed surface applicator.

**Figure 8:**
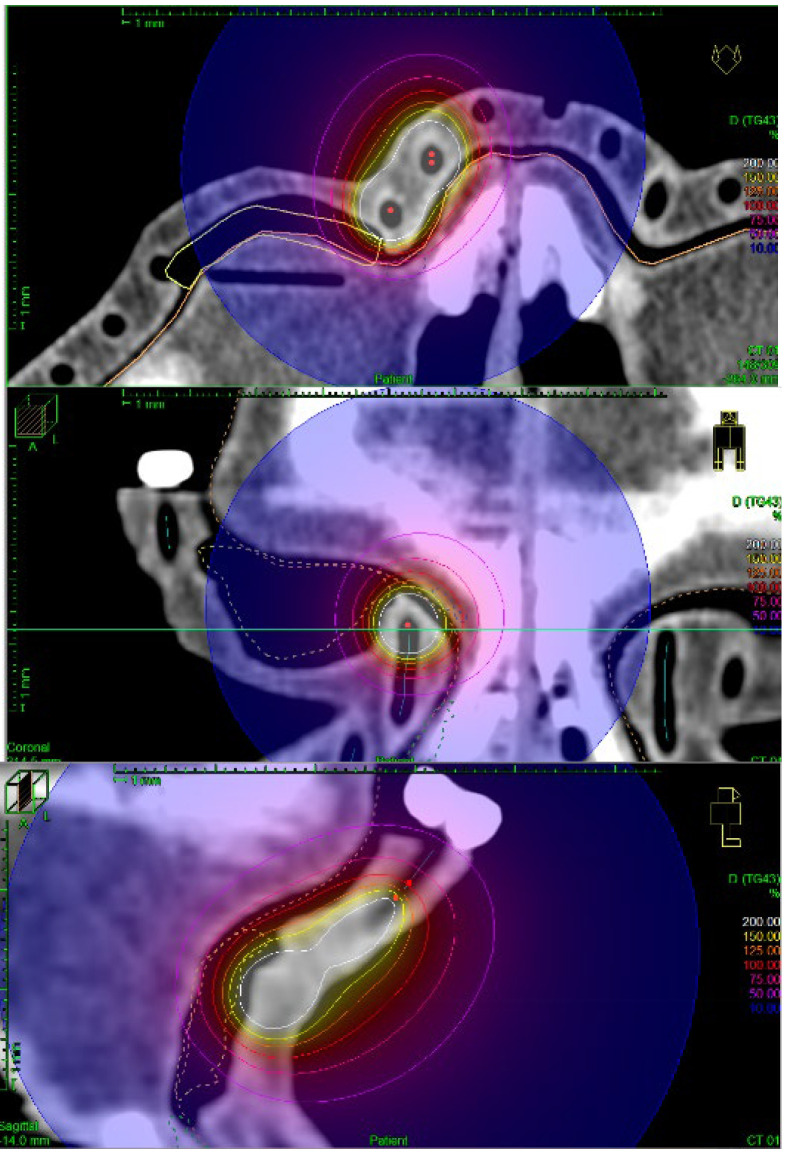
Detailed view of sample isodoses obtained during the treatment planning process; the optimization of the treatment plans.

The treatment planning process was based on determining the dose prescription points (patient points) in the TPS at the border of the target areas. It was possible to perform preliminary normalization and optimization of the plan on the designated points. To improve the automatically generated plan, manual normalization, and graphical optimization processes were performed, which improved the dose distribution near the target volumes. The prescribed dose in each plan was 10 Gy. The step distance between each source stop position was set as 1 mm. Each treatment plan was recalculated in several ways. First, the TG-43 formalism, which is recommended for use in brachytherapy planning procedures, was used. TG-186 formalism was used as a second, which took into account the presence of lead shielding and the scattering resulting from the use of a PLA applicator. The TG-186 formalism was used in two ways in standard (TG186) and high accuracy (TG186H) modes. An example of the obtained isodose distribution for the treatment plan realized by the authors is shown in Figure 7 and Figure 8.

**Figure 9:**
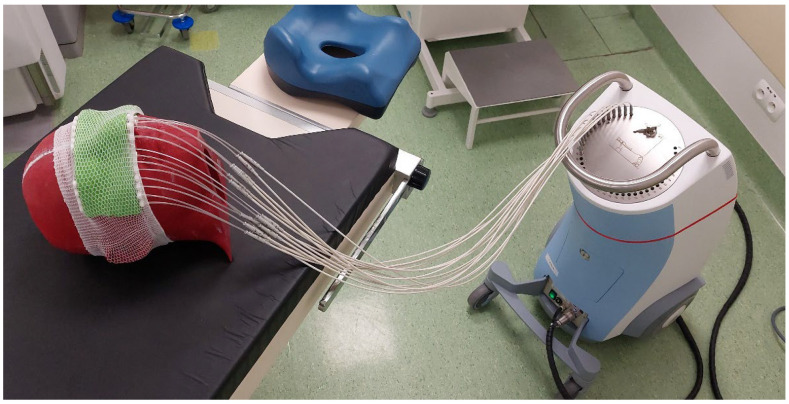
Final setup for performing the measurements with phantom, applicator, and Ir-192 afterloader (Flexitron^TM^ by Elekta^TM^).

**Figure 10:**
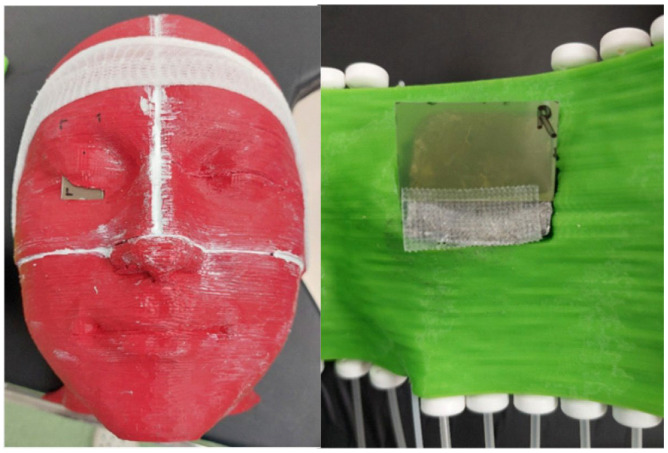
Gafchromic EBT3 film detectors. On the left side, the area of the eye lens, on the right side, the area of the eyelid surface.

Each of the prepared treatment plans was carried out twice; with and without the lead shield on the eye (a missing applicator piece made of PLA was placed in its place). A properly cut Gafchromic EBT3 radiochromic film was used to measure the doses for each plan. During irradiation (Figure 9), one of the film detectors was located in the pocket prepared in the right eye. The other film detector was placed on the surface of the right eye, directly under the shield as shown in Figure 10.

To obtain the data, the irradiated films were digitized using the Epson Perfection^TM^ V750 scanner in the same orientation they were irradiated. Films were digitized according to the order of irradiation. All films were placed in the center of the preheated scanner, in the same orientation, with image correction functions disabled, using the same scanning parameters, with a resolution of 72 dpi and 48-bit color depth. After digitizing—the files carried information about the ADC (analog to digital conversion) signal collected by the scanner, were saved in lossless TIFF format end exported to OmniPro I’mRT ^TM^ software. To properly calibrate the data, a calibration curve determined for the work of Bieleda et al. [8] was used. This made it possible to convert the data and read out information about the absorbed dose. Film detectors always require a calibration process to obtain the measurements, due to their nonlinear dose response. Dose readouts of the films were performed in a point-wise manner. Readings from 10 points were averaged.

## 3. Results

### 3.1. Doses at Critical Organs and Target Areas Calculated Using Treatment Planning System (TPS) Preliminary Data and Calculation Engine Analysis

The doses deposited in a 0.1cm^3^ volume of critical organs and film slit (LENS_0.1_; EYE_0.1_; BONE_0.1_; FILM_0.1_), the dose deposited in a 2 cm^3^ volume of the eye (EYE_2_), and the values of the D_90_, V_100_ and V_150_ parameters are summarized in Table 1. Doses were calculated three times: using the TG43 formalism (no shielding), using the TG186 formalism (with standard accuracy), and TG186H (with high accuracy), which took into account the use of a lead shield. The values presented in the tables show the percentage fraction of the prescribed dose which for all ten plans was set as 10 Gy. The calculations were performed for all analyzed treatment plans (CTV 1–CTV 10).

### 3.2. Measurements on the Skin Surface (Eyelid)

The results obtained from radiochromic films placed on the surface of the eye during lead-shielded irradiation are shown in Table 2. The parameter D_n_ denotes the dose deposited from the n-th plan (for CTV 1 to CTV 10).

The results obtained from reading the doses deposited on radiochromic films placed on the surface of the eye during irradiation without a lead shielding are shown in Table 3.

**Figure 11:**
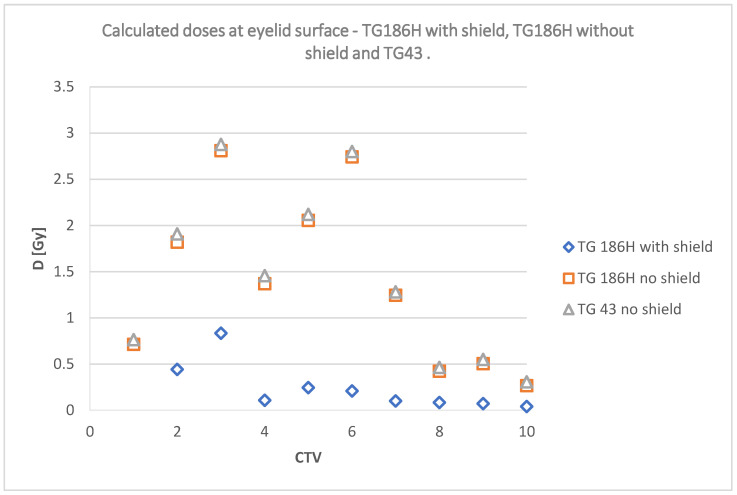
Calculated doses comparison—TG186H with shield, TG186H without shield, and TG43 on the eyelid surface of the right eye.

The chart below (Figure 11) shows a comparison of the calculated doses on the eyelid surface without a shielding according to the TG43 and TG186H formalisms and with a shield according to the TG186H formalism.

All the measurements made, and the results from the TPS calculations for a point on the surface of the eyelid were collected in Table 4.

**Figure 12:**
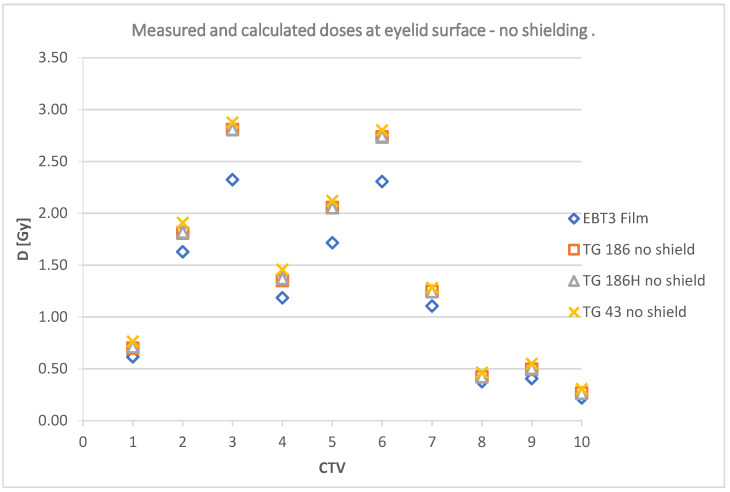
Deposited doses measured at a point on the surface of the eyelid without a shielding.

**Figure 13:**
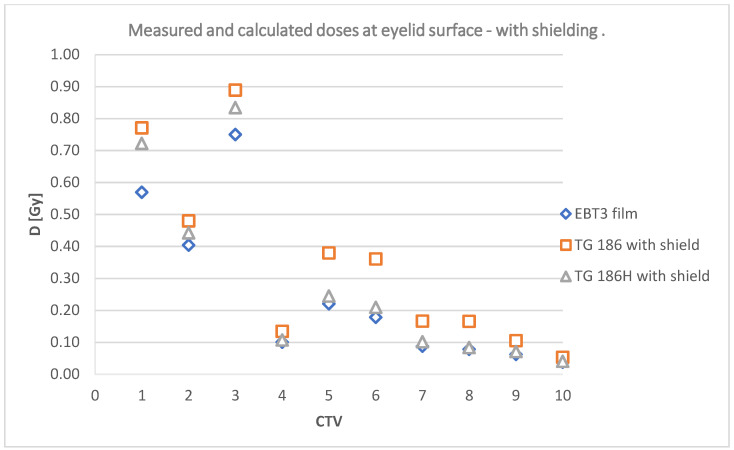
Deposited doses measured at a point on the surface of the eyelid using the lead shielding.

The data collected in Table 4 are shown graphically in Figure 12 and Figure 13; and include a summary of dose values for the corresponding plans using TG43, TG186, and TG186H formalisms, as well as doses measured using radiochromic films without shielding and TG186, TG186H and films with shielding, respectively.

### 3.3. Measurements of the Dose in the Lens of the Eye

The results obtained from radiochromic films placed at the lens region, for lead-shielded irradiation are shown in Table 5.

The results obtained from radiochromic films placed at the lens region during irradiation without lead shielding are shown in Table 6.

Dose values measured by films placed in the lens region indicate that the use of lead shielding has a beneficial effect on the protection of this organ. The averaged doses at the analyzed point (lens) differ significantly on the presence or absence of the lead shielding.

**Figure 14:**
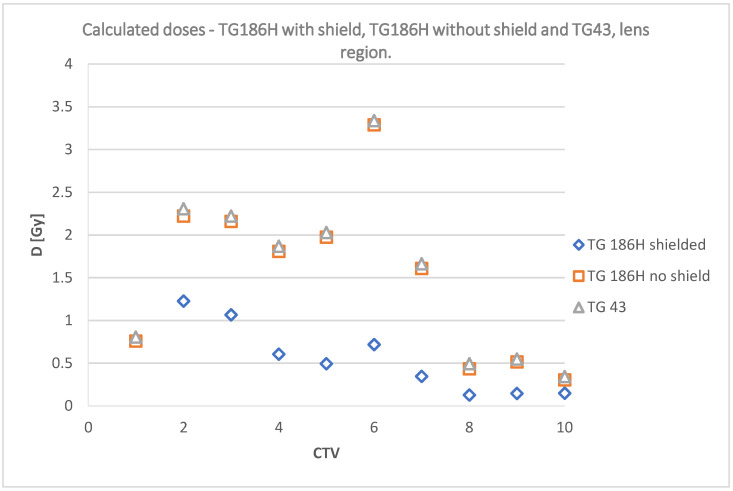
Calculated dose comparison—TG186H with shield, TG186H without shield, and TG43 at a point in the lens of the eye.

TPS calculated dose values at the point corresponding to the center of the lens were also analyzed depending on the use of the TG186 high-accuracy or TG43 formalism. The observed dose differences are shown in Figure 14.

Table 7 shows the doses in the lens determined during measurements using radiochromic film with and without lead shielding, as well as the doses calculated in the treatment planning system for TG43, TG186, and TG186H formalisms with and without the shielding.

**Figure 15:**
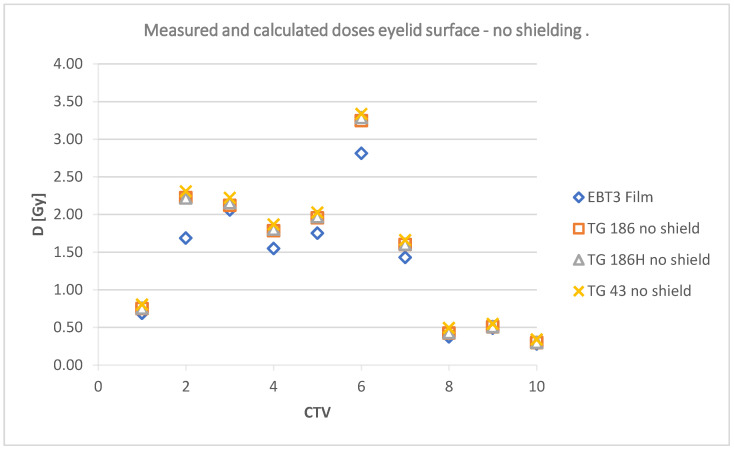
Doses at a point in the center of the lens of the eye without a shielding.

**Figure 16:**
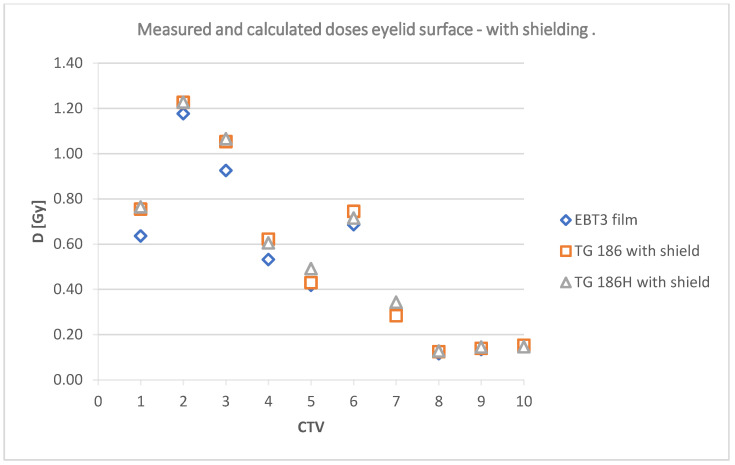
Doses at a point in the center of the lens of the eye with a shield.

The doses at a point in the center of the lens calculated in TPS and measured using film for irradiation without shielding are shown in Figure 15. Figure 16 shows the doses in the lens for the analyzed cases with shielding included.

### 3.4. Statistical Analysis of the Results

To analyze the results, a *t*-test was used to compare single averages. Before performing the analysis, the measurement data were checked for compliance with the normal distribution using the Shapiro–Wilk test. The measurements show normal distribution at the level of α = 0.05.

### 3.5. Eyelid Surface

For the shielded plans the calculated values, using TG186 formalism, did not show statistical compliance in any of the analyzed cases (*p*-values 0.0 to 0.01). On the other hand, the analysis of the TG186H calculated values vs. Gafchromic^TM^ film measurements showed no statistically significant differences for plans CTV 2, 4, 5, 8, and 10. The high-accuracy algorithm proved to be more appropriate for the calculation of the dose distribution with lead shielding. Mean dose values from TG186 were significantly overestimated compared to measurements with the radiochromic film. On the other hand, the average readings from the films differed from the dose values calculated with the use of TG186H mode. This trend was not related to the different locations of the target volumes.

For the data obtained without lead shielding for CTV 1 region, agreement was observed for TG186 (*p* = 0.054), but not confirmed by TG186H (*p* = 0.03). However, the small difference at the used level of significance indicates that the small discrepancies that occur when using two levels of calculation accuracy were preserved.

There were no statistically significant differences observed for both TG186 (sequentially for plans 7 and 8: *p* = 0.052, *p* = 0.55) and TG 186H (*p* = 0.146, *p* = 0.143). The values measured by Gafchromic^TM^ film for these three plans matched the calculations made in TPS using the algorithms suggested by TG186 formalism.

TG43 calculations showed no agreement with any of the film measured doses from the plans analyzed (*p*-values ranging from 0.0 to 0.02). The values indicated by TG43 in each of the analyzed plans were higher than those of the TG186 formalism and the film readings. The average values measured by the films were lower than the values suggested by TG43 calculations.

### 3.6. Lens Region

Doses measured by Gafchromic EBT3 films for treatment plans with lead shielding were found to be statistically consistent with the TG186 formalism in 7 out of 10 cases (CTV 2, 5, 6, 7, 8, 9, 10). In the case of CTV 5, however, the TG186H high-accuracy mode did not show compliance (*p* = 0.023), for the other plans there were no statistically significant differences observed for the TG186H vs. TG186 mode of the calculation engine. The average readings from the films turned out to be close to the results proposed by TPS and taking into account the presence of lead shielding in 5 out of 10 plans. On the other hand, for plans CTV 1, 2, 3, 4, and 6, the dose values measured by the films were lower than those calculated by the treatment planning system.

For the unshielded plans, the average doses measured by the films did not agree with the doses calculated using TG43 formalism, except for plan CTV 3 (*p* = 0.107). The TG186-based calculations assuming no shielding showed no statistically significant differences for plans CTV 1, 3, 6, 8, 9, and CTV 10. The high-accuracy mode (TG186H) showed agreement with exception of plan CTV 6 (*p* = 0.042).

For the calculated doses in the lens region, without the use of a lead shield, the TG186H calculation algorithm proved to be the closest to the measured data. There were no statistically significant differences observed between the dose readings from the films and the calculated doses (*p* = 0.058 to 0.408). On the other hand, dose measurements showed a lower dose than the dose calculated by the corresponding formalisms in the treatment planning system.

## 4. Discussion

The purpose of this study was to verify whether the lead shielding may increase the dose deposited in the eyelid surface and lens of the eye during superficial brachytherapy procedures. The basic idea was to observe an increased dose for irradiation with a shield than without one. It was also necessary to design a suitable measurement system to measure the dose deposited in the lens of the eye.

The AAPM TG43 formalism accurately describes the effect of source geometry but does not take into account the effect of inhomogeneities in the medium. As the answer to this problem, TG-186 was proposed. Model-based dose calculation algorithms (MBDCA) can be used to improve the accuracy of dose calculations. The AAPM 186 Task Group (TG186) issued guidelines for the use of these algorithms [9]. One of the dose calculation methods is Advanced Collapsed Cone Engine (ACE) dose calculation model. ACE uses the principle of PSS (The Primary and Scatter Separation), which assumes a superposition of multiple scatters (or successive scatters) to improve the accuracy of scattered dose calculations. The dose is divided into a primary component, a first scattering component, and a multiple scattering component. Each dose component is calculated using pre-parameterized dose distributions generated from Monte Carlo simulations in water for a particular source of irradiation. The primary dose is calculated using the ray-tracing method to take into account materials with densities other than the water along the radiation path, while the scattered dose components are calculated using the collapsed cone superposition method.

The primary dose, due to the short range of secondary electrons, depends only on the density of the material along the radiation path between the source and the area of dose deposition. The scattered dose depends both on the direct irradiation pattern and on the distribution of physical properties of structures in the large volume surrounding the point of interest, i.e., a much larger volume must be included in the calculation to integrate many contributions to the final doses. This method uses an order-controlled scattering process with a discrete number of transport paths determined by tessellating a spherical surface centered at the point of interest. The number of transport paths is dictated by the accuracy mode selected in the TPS (standard or high). To calculate dose in a medium other than water, creating contours of structures in CT cross-sections and assigning them a tissue type from the material library is also possible. Electron densities can be determined in two ways: by assigning a uniform density specified by the selected material (listed in Table 3 of the TG186 report), or by using individual voxels to convert Hounsfield unit (HU) values to electron densities [10,11,12,13].

A number of different dosimetry tools can be used to verify treatment plans generated by treatment planning systems. One of the groups is radiochromic film detectors. These detectors take advantage of the chemical transformations occurring in the materials under the influence of radiation. GAFChromic® EBT-3 radiochromic films were used to measure the absorbed doses for this study. They consist of a 28 µm active layer sandwiched between two 125 µm thick matte–polyester substrates. The active layer contains an active component, a marker dye, stabilizers, and other components that give the film an energy-independent response. The dose measurement range of the films is 0.2 to 10 Gy, which makes the films suitable for dosimetry in brachytherapy. They are characterized by high spatial resolution, which is an advantage during dosimetric verification of dose distributions with a high gradient, as in the case of HDR brachytherapy. These film dosimeters do not exhibit angular dependence, allowing for measuring the full in-plane dose distribution during a single exposure. Under controlled conditions, the results obtained from the measurements are reproducible with an accuracy of 2-3%. In addition, the films are waterproof and thus suitable for use in a water phantom, also easy to shape by simple trimming. They are energy-dependent in the range below 100 keV, which is a certain limitation of this dosimetry method. Film dosimeters do not require post-exposure processing, and there is no need to use a darkroom when working with films. However, they are designed for use in a lighted room; exposure to direct sunlight should be avoided. Measurements should be taken at room temperature (20–25 °C), but films should be stored in a refrigerator [14,15].

Proper verification of deposited doses was strongly dependent on the production batch/series of films used, as well as the scanner itself, so calibration had to be performed before evaluating deposited doses using the designed applicators. The calibration curve used in the study by Bieleda et al., was used for calibration purposes for this study [8]. Film dosimeters seemed to be the optimal solution, due to their wide dose range (according to the manufacturer’s recommendations, measurements in the range of 0.2–10 Gy), as well as easy adaptation to the measurement system through the possibility of clipping. A study by Chiu-Tsao S. et al., showed that the films are energy independent at doses above 1 Gy and photon energies greater than 100 keV [16]. EBT3 dosimeters can therefore be used for 192Ir sources with an average photon energy of 380 keV.

For contact brachytherapy of the facial region, applicators prepared individually for the patient have several advantages. They provide repeatable source positioning over irradiation sessions. Repeatability of source positioning is particularly important, due to the high dose gradients that occur with HDR brachytherapy. They also allow the applicator to precisely match the anatomical curves of the face, especially in the region of the ears, eyes, or nose. They are prepared according to the patient’s CT images, from materials such as ABS (Acrylonitrile Butadiene Styrene copolymer) or PLA. These materials are non-toxic and readily available. Applicators are created using free software and a treatment planning system available in the treatment center [2]. PLA polylactide is a material formed by heating lactic acid. Due to its high manufacturing cost, it was initially used mainly in medical applications. However, after the development of technology to produce polylactide by glucose fermentation, production costs decreased, and the use of PLA became more widespread. Currently, PLA is obtained from corn meal or sugar cane, making it a biodegradable material. PLA is a linear aliphatic polymer. It is characterized by high transparency and gloss, high rigidity, and ease of molding. It has a relatively high density (1.25 g/cm^3^) and a low melting point of 173 °C. Its widespread use for the purpose of 3D printing is determined by good thermoplastic properties and low processing shrinkage. When realizing FDM printing (Fused Deposition Modeling), the temperature of the printer hot end is usually set as 175–235 °C. The filament is used to create intricate parts and details, and due to its low price and lack of harmful effects on the environment, it is also popular for printing prototypes. However, the material has its drawbacks. PLA is a hygroscopic material and is not resistant to UV radiation. Therefore, should be stored in a shaded area with low humidity. A low melting point also makes it sensitive to high temperatures. Compared to other fibers, it is inflexible and brittle [17]. A PLA print with 90% infill on the HU (Hounsfield Units) scale is close to water (range -36 to 44 HU), depending on X-ray energy. Decreasing the infill of the printed details causes the density of the objects to decrease. However, due to the low cost of PLA material, reducing the infill is rather not necessary [2,3].

### 4.1. Doses at Critical Organs and Target Areas Calculated Using Treatment Planning System (TPS)

A separate treatment plan was prepared for each of the designated target areas, taking into account the dose coverage of a specific CTV only. In the evaluation of brachytherapy treatment plans, the maximum dose D_max_ was not used, the parameter D_0.1_, denoting the dose deposited in 0.1 cm^3^ volume of the area of interest has been taken into account. Due to the high dose gradients observed in brachytherapy treatment, D_0.1_ is a more valuable parameter for treatment plan verification. Other important values to consider are the values of D_90_, V_100,_ and V_150_. The D_90_ represents the percentage dose deposited in 90% of the volume of the organ. The parameters V_100_ and V_150_ represent the percentage volume of the organ covered by isodoses of 100% and 150% of the prescribed dose, respectively.

A comparison of the doses calculated in 1cm^3^ of the lens when using the TG43 formalism and TG186, which takes into account the use of a lead shield, clearly shows that the use of shields reduces the dose by several times. Depending on the location of the target area, the dose calculated in the lens when a shield is used is equal to or much lower than without a shield. In the case of CTV 7 (inner corner of the eye, bottom), the use of a shield reduced the calculated dose by more than five times. Plans prepared for the other target areas located near the right eye (CTV 2–CTV 6) recalculated with lead shielding also showed that the dose in the lens is significantly lower. For the location of the target area on the right temple, on the tip of the nose, and near the left eye, the use of a shield made little difference to the calculated doses. The dose was reduced slightly to within a dozen percent. These locations of the target areas corresponded to the situation when the shield was in front of the geometric line between the lens and the source positions.

In the case where the target was located at the top of the eye or on the opposite side of the face than the considered critical organs, the treatment planning system did not register the dose in 0.1cm^3^ of the lens with the shield. In this case, the analyzed parameter was the dose deposited in 1cm^3^ and 2cm^3^ of the eye. The use of the shield made the dose decrease by about 1% compared to the situation without using it. In none of the analyzed cases the dose in the eye and lens increases, as a result of the application of the lead shield. Similar results to those of the LENS_0.1_ and the EYE_0.1_ and EYE_2_ were shown by the FILM_0.1_ parameter. In each of the analyzed cases, the dose calculated in these volumes decreased when a lead shield was applied to the eye region, or the shield had no significant effect on the calculated doses. The use of shields also affected the dose in the bones (BONE_0.1_). The dose was slightly reduced or the shielding did not significantly alter the dose.

The V_100_ parameter is considered acceptable when the volume that receives a dose equal to 100% of the prescribed target dose is equal to or higher than 90%. V_150_ is acceptable when a dose of 150% is deposited in a volume less than 25%, while D_90_ is above 100%, meaning that 90% of the target volume receives a dose equal to 100% of the target dose. For all of the above plans, it was possible to keep the D_90_ and V_100_ parameters consistent. However, due to the rather problematic location of the target volumes and the shape of the designed applicator, it was impossible to maintain the optimal level of the V_150_ parameter. It was necessary to find a compromise between optimal coverage of the target area with the prescribed dose and prevention of so-called “hot spots”. Excessively large distances between the catheters and the target areas and the design of the applicator caused the V_150_ parameter to be exceeded in 3 out of 10 cases.

The use of a lead shield over the eye did not affect the calculated V_100_, V_150,_ and D_90_ parameters. The 1–2% differences in doses were observed due to the difference in the calculation algorithms of the TG43 and TG186 formalisms with standard and high calculation accuracy.

### 4.2. Measurements on the Skin Surface (Eyelid)

The obtained data indicate that the doses without a shield were higher than those measured with a lead shielding. Regardless of the location of the target volume, the use of shielding resulted in lower average dose readings at a point on the surface in each of the prepared plans. The data obtained from the films were compared to the doses calculated in the treatment planning system at the same points. TG43 and TG186H formalisms with and without consideration of the shielding were used. The applicator material (PLA) was estimated in TPS as Plastic Water^TM^, a material with a density of 1.006 g/cm^3^.

The relationship shown in the Figure 11 indicates that, according to TPS calculations, the use of a shield reduces dose in 9 out of 10 cases, excluding the target volume located on the right temple. For the plans with CTV located near the left eye, the dose is only slightly lower. A large difference in doses is predicted by the system for target areas located near the right eye, the most for a plan with the irradiated lesion in the inner corner of the eye, right next to the shield. The use of shielding in cases CTV 2–CTV 7 allows the calculated dose reduction by up to three times. Doses calculated using the TG43 formalism are very close to those calculated by TG186H. However, the tendency of TG43 to slightly overestimate the dose relative to the TG186H formalism was observed.

### 4.3. Measurements of the Dose in the Lens of the Eye

The relationship shown in the Figure 14 indicates that, according to TPS calculations, the use of a shield reduces dose in 9 out of 10 cases, excluding the target located on the temple. The shield did not affect the dose deposited in the lens during plan CTV 1. For plans with CTV regions located on the tip of the nose (CTV 8) and near the left eye (CTV 9 and 10), the dose is slightly lower. A large difference in dose is predicted by the system for targets located near the right eye, mostly, for a plan with irradiated lesion at the inner corner of the eye, right next to the shield (CTV 6). Similar to the point on the surface of the eye, the TG43 formalism indicates a slightly higher dose value than TG186H.

Figure 15 and Figure 16 suggest that the radiochromic films indicate lower doses than the TPS calculation algorithms. The results are particularly different for CTV 2, 4, 5, 6, and CTV 7 without shielding. These target areas were located in the inner corner of the eye and under the eye. With a different geometry (source vs. lens vs. shield), these doses did not differ significantly. The use of a lead shield affected the obtained dose values. With shielding included, the films indicated a lower dose in five target areas (1, 2, 3, 4, and 6). In the remaining plans, the film reading was equal to or higher (CTV 7) than the values reported by TG186 and TG186H.

For superficial brachytherapy in the head region, an important consideration is the protection of the organ of vision, especially the lens of the eye. HDR brachytherapy treatments are characterized by a very high dose gradient, which means that even a small displacement of the applicator can cause large differences in dose distributions. The use of lead shielding is a common clinical practice, especially for lesions located near the eye. Due to its physical properties (density, atomic number) lead is the material of choice in this case. In addition, lead sheet of sufficient thickness is flexible enough to conveniently shape the required curvature without the use of specialized tools. The use of other materials for the preparation of shielding would be less effective due to physical properties. Lead is a widely used and available material used in radiotherapy (except for Wood’s alloy). In the case when surface applicator is prepared by stereolithography, the use of lead sheet allows to prepare shielding with dimensions that do not significantly interfere with the geometry of the applicator, geometry of treated region and at the same time allow the required radiation attenuation. However, there is a lack of published reports considering the possible negative effect of the shielding on the doses deposited in the lens when the lesion being irradiated is located linearly behind the shield. The geometry when the shield is not located between the radiation source and the critical organ may affect in an unwanted dose deposition in the critical organ from secondary radiation caused by the interaction of ionizing radiation from the brachytherapy source with the shield material.

The anthropomorphic phantoms available on the market do not allow measurement of the exact region of the lens of the eye. Water-box phantoms, on the other hand, do not reflect the actual clinical situation. To maximize similarity to a real treatment procedure, it was decided to design and create an anthropomorphic phantom by stereolithography method that would allow dose measurements at the exact planned point in the eye. The phantom itself and applicator were printed from PLA, which is recognized as a safe thermoplastic material that can be used in both brachytherapy and teleradiotherapy. PLA was also chosen as the material for creating the phantom because it is readily available, and at the same time mimics soft tissue very well in CT scans, as was demonstrated in the work by Marchant Van der Walt et al. They analyzed PLA samples in terms of Hounsfield units (HU) to determine relative electron density (RED) and mass density, and then compared them to several commercial tissue phantoms with different properties, as well as strength tests for radiation damage [18]. O.L. Dancewicz et al. also found PLA to be a suitable material for use in radiation therapy [19].

Analysis of dose measurements using dosimetry films and doses calculated by the treatment planning system showed that the use of shielding in most cases has a beneficial effect on dose reduction in critical organs. Doses deposited under shielding during irradiation of target areas in the immediate vicinity of the eye appeared to be 3–5 times lower than without the use of shielding. Smaller dose reductions were observed when the shield was not between the radiation source and the critical organ. When the shield was in front of the line connecting the lens and the source position, the doses were higher than expected. The geometry of the lens-shield system appeared to influence the obtained results. However, no trend was observed, and dose values obtained from the dosimetry films were lower or higher, regardless of the position of the source path relative to the shield.

The material of choice for most radiation shielding is lead with an atomic number Z equal to 82. The energy range used in brachytherapy (0.3–0.9 MeV) during interaction with lead shielding may result in the occurrence of primarily the photoelectric effect, as well as the Compton scattering. The electron–positon pair formation for materials with such high Z occurs for energies higher than 1 MeV. The effects of megavoltage and kilovoltage radiation on lead shielding and the resulting backscattered radiation were analyzed in several other papers [20,21,22]. They showed that backscattered electrons and photons are recorded when interacting with lead shielding with generated radiation energies of several hundred keV. Thus, the range of backscattered radiation reaches several millimeters. The energy of the backscattered radiation is lower than the primary radiation. For a 192Ir source placed in water, Candela et al. observed that the average energy of photons and electrons emitted in the backward direction (defined as the direction from the shielding to the source) was lower than in the forward direction, and also decreased as the distance from the source increases, also the average energy of photons and electrons passing through the lead shielding decreased slightly with increasing distance from the source [23]. Short-range backscattered radiation appears on the surface of the shielding material. Backscattered radiation from lead shielding has been partially recorded by dosimetry films, as evidenced by discrepancies in the results obtained from the analysis. The energy of the resulting radiation is probably lower than the energy range of the film. Radiochromic films, however, are energy-dependent for energies lower than 100 keV, which caused inaccuracies in the readings and failure to register the total dose deposited under the shielding. The backscattered radiation has a range of a few millimeters thus some of the radiation was detected at a point on the surface of the eyelid just below the shielding.

The dose measurements by the radiochromic film may also have been influenced by the distance of the film from the source. Backscattered radiation has a range of several millimeters in water. However, this range decreases in air. Much of the radiation may therefore have already dissipated in the applicator material, as well as in the air space between the shielding and the film. Radiation scattering was also affected by the type of phantom. Radiation scattering also occurred in the air spaces and bones simulated in the phantom. The low-energy and short-range backscattered radiation that may have been produced on the lead shield as a result of the irradiation was not detected in its entirety on the films.

The first-choice dosimetric method for brachytherapy using low-energy sources remains LiF TLD detectors [24]. Due to the high dose gradient observed in HDR brachytherapy, this method may be not optimal. Developing a reliable dosimetric system for high-dose brachytherapy is a rather challenging task. The use of radiochromic films proved to be the optimal solution for use in the present study.

Dose measurements using radiochromic film have been shown as obviously less consistent than the calculation methods proposed by TG186. The calculation methods proposed by TG186 are based on the division of the total dose into primary dose, single, and multiple scattered one. This assumption proved to be valid when analyzing the lead-shielded measurement system. Dose calculation algorithms, taking into account the presence of a shield with a density of 11.3 g/cm^3^ in the radiation range, gave a dose corrected for the value of the dose deposited by radiation backscattered on the lead. Both the standard-accuracy algorithm and high-accuracy algorithm indicated a significant contribution to the total dose from the dose deposited by backscattered radiation. The TG186 calculation methods also changed the distribution of isodoses in the presence of lead shielding, respectively. However, TG186 formalism with its high accuracy in calculations without consideration of lead shielding gave similar results to TG43. This indicates that accounting for multiple scattering when there are no high atomic number materials near the source does not change the dose distributions proposed by TG43.

The formalism proposed by TG186 is more adequate in the situation when shields are used. TG43’s calculation methods assume uniform radiation scattering in a water phantom of infinite volume. Therefore, they cannot be used as a reference for the real clinical situation with lead shielding. In a clinical situation, it is also not possible to assume full scattering conditions (air gaps, area of interest near the skin–air border). Due to the negligible contribution of backscattering in the air compared to backscattering in water, the calculated surface dose proposed by TG43 may currently be overestimated. TG43’s methods do not take into account the physical densities of objects surrounding the source, which affect the dose distribution. However, when high Z materials are used in close proximity to critical organs or target areas, the dose distribution may change. When a dose deposition due to backscattered radiation is probable, it seems optimal to compare TG43 calculations with TG186.

The inconsistency of the results obtained, and the lack of an increasing or decreasing trend depending on the position of the shield relative to the lens leads to the personalization of the treatment procedure. For this purpose, it would be necessary to prepare dummy-shields from tissue-like material (e.g., printed independently in a 3D printer from PLA material). The dummy-shields would have to be taken into account during CT imaging for treatment planning. Using TG186 methods, it would be necessary to determine the density of the imaged artificial shield as uniform lead with a density of 11.3 g/cm^3^ and then perform the dose distribution calculations. After taking into account the dose distribution from the TG186 and TG43 algorithms, a decision would have to be made on whether or not to use lead shielding during treatment.

## 5. Conclusions

1) A low-cost fabrication procedure for an anthropomorphic phantom using 3D-printing technology was developed. The phantom, previously designed in the OncentraBrachy^TM^ treatment planning system, was printed in the FDM technique using PLA polymer;
2) A radiochromic film-based method was developed, to measure the dose deposited in the lens of the eye and at a point on the surface of the eyelid directly under the lead shield using an individual surface applicator;
3) Doses calculated in the treatment planning system were compared with doses measured using radiochromic films for the personalized surface brachytherapy applicator. The differences observed are the consequence of the inability of this method to record short-range, low-energy radiation backscattered from the shielding material;
4) Comparing the doses planned in the treatment planning system, using the TG43 formalism (full scattering in water), and the TG186 formalism, which takes into account the electron densities of the surrounding matter and the geometry of the irradiated system, it can be assumed that the use of lead shields is a method for protecting the organ of vision from the adverse effects of ionizing radiation. Lead shields are especially important for protecting the highly radiation-sensitive lens of the eye. However, the decision to use a lead shield during facial surface brachytherapy procedures should be made on a patient-by-patient basis and based on model-based calculation methods recommended by TG186.

## Figures and Tables

**Table 1:** Calculated dose-distribution parameters for analyzed volumes of critical organs (OAR) and CTV (1–10) volumes. TG 43—without shielding, TG186, TG186H—with lead shielding.

OAR	TG 43 [%]	TG186 [%]	TG186H [%]	OAR	TG 43 [%]	TG186 [%]	TG186H [%]
LENS_0.1_	6.06	5.7	5.73	LENS_0.1_	17.44	8.85	8.83
EYE_0.1_	18.97	18.02	18.17	EYE_0.1_	49.79	42.04	42.41
EYE_2_	11.87	11.35	11.41	EYE_2_	25.65	19.58	19.79
BONE_0.1_	67.65	64.85	64.05	BONE_0.1_	63.71	62.03	61.71
FILM_0.1_	11.00	10.28	10.46	FILM_0.1_	32.33	16.52	16.39
**CTV 1**				**CTV 2**			
D_90_	102.34	102.01	102.2	D_90_	101.53	102.65	102.38
V_100_	94.05	93.5	93.55	V_100_	91.89	93.09	92.78
V_150_	3.69	2.92	2.56	V_150_	17.97	18.59	18.19
**OAR**	**TG 43 [%]**	**TG186 [%]**	**TG186H [%]**	**OAR**	**TG 43 [%]**	**TG186 [%]**	**TG186H [%]**
LENS_0.1_	17.16	8.35	8.43	LENS_0.1_	14.09	3.53	3.38
EYE_0.1_	50.66	44.95	45.01	EYE_0.1_	33.48	25.39	27.07
EYE_2_	25.99	20.48	20.42	EYE_2_	18.59	11.24	12.15
BONE_0.1_	54.08	52.08	51.56	BONE_0.1_	67.65	65.04	65.65
FILM_0.1_	37.27	20.24	20.36	FILM_0.1_	26.74	9.72	9.6
**CTV 3**				**CTV 4**			
D_90_	112.68	113.96	113.78	D_90_	101.13	102.04	101.66
V_100_	98.55	98.82	98.82	V_100_	90.68	91.17	90.99
V_150_	8.52	8.3	7.97	V_150_	49.47	50.23	49.91
**OAR**	**TG 43 [%]**	**TG186 [%]**	**TG186H [%]**	**OAR**	**TG 43 [%]**	**TG186 [%]**	**TG186H [%]**
LENS_0.1_	19.28	3.6	3.66	LENS_0.1_	31.22	6.07	5.77
EYE_0.1_	40.1	26.55	28.03	EYE_0.1_	66.67	37.5	37.33
EYE_2_	20.04	10.99	11.04	EYE_2_	31.15	13.94	13.01
BONE_0.1_	67.53	66.32	66.28	BONE_0.1_	92.08	89.56	88.85
FILM_0.1_	25.11	8.82	7.82	FILM_0.1_	42.86	9.7	9.5
**CTV 5**				**CTV 6**			
D_90_	111.76	113.12	112.93	D_90_	102.91	105.03	104.73
V_100_	96.43	96.94	96.85	V_100_	94	96.31	95.96
V_150_	15.6	17.55	17.08	V_150_	23.56	26.57	26.18
**OAR**	**TG 43 [%]**	**TG186 [%]**	**TG186H [%]**	**OAR**	**TG 43 [%]**	**TG186 [%]**	**TG186H [%]**
LENS_0.1_	14.46	2.52	2.54	LENS_0.1_	3.56	-	-
EYE_0.1_	28.38	13.73	14.71	EYE_0.1_	9.87	7.55	7.65
EYE_2_	15.2	5.51	5.78	EYE_2_	5.66	3.15	3.26
BONE_0.1_	95.06	92.08	91.41	BONE_0.1_	26.53	25.11	25.11
FILM_0.1_	20.77	4.47	4.84	FILM_0.1_	6.15	-	-
**CTV 7**				**CTV 8**			
D_90_	102.25	103.35	103.46	D_90_	102.83	102.07	102.06
V_100_	91.48	92.16	92.16	V_100_	94.31	93.25	93.22
V_150_	43.67	44.38	44.34	V_150_	11.21	10.38	10.28
**OAR**	**TG 43 [%]**	**TG186 [%]**	**TG186H [%]**	**V OAR**	**TG 43 [%]**	**TG186 [%]**	**TG186H [%]**
LENS_0.1_	4.12	-	-	LENS_0.1_	2.52	-	-
EYE_0.1_	11.3	9.97	9.98	EYE_0.1_	9.38	8.76	8.76
EYE_2_	6.86	5.69	5.7	EYE_2_	5.06	4.29	4.28
BONE_0.1_	67.12	66.08	63.41	BONE_0.1_	54.06	53.04	51.65
FILM_0.1_	7.21	4.5	4.49	FILM_0.1_	5.03	3.19	3.18
**CTV 9**				**CTV 10**			
D_90_	104.05	104.24	104.05	D_90_	102.77	103.12	102.82
V_100_	92.87	92.98	92.89	V_100_	94.26	94.75	94.32
V_150_	22.53	22.44	22.29	V_150_	17.66	17.53	17.08

**Table 2:** Doses are measured at a point on the surface of the eye (eyelid) under the lead shield.

POINT NO.	D_1_ [Gy]	D_2_ [Gy]	D_3_ [Gy]	D_4_ [Gy]	D_5_ [Gy]	D_6_ [Gy]	D_7_ [Gy]	D_8_ [Gy]	D_9_ [Gy]	D_10_ [Gy]
1	0.48	0.31	0.85	0.11	0.26	0.13	0.08	0.08	0.05	0.04
2	0.44	0.50	0.83	0.11	0.27	0.18	0.07	0.06	0.08	0.04
3	0.51	0.46	0.69	0.11	0.23	0.16	0.10	0.07	0.06	0.04
4	0.61	0.49	0.53	0.07	0.20	0.22	0.09	0.08	0.06	0.03
5	0.48	0.41	0.81	0.12	0.15	0.14	0.10	0.09	0.04	0.03
6	0.68	0.30	0.83	0.11	0.26	0.18	0.08	0.09	0.07	0.04
7	0.45	0.31	0.80	0.11	0.15	0.15	0.07	0.09	0.07	0.04
8	0.56	0.45	0.71	0.10	0.20	0.22	0.09	0.08	0.06	0.04
9	0.69	0.41	0.81	0.06	0.22	0.23	0.10	0.08	0.07	0.03
10	0.82	0.41	0.64	0.10	0.27	0.16	0.11	0.07	0.06	0.04
**MEAN**	0.57	0.40	0.75	0.10	0.22	0.18	0.09	0.08	0.06	0.04
**STD DEV.**	0.12	0.07	0.10	0.02	0.04	0.03	0.01	0.01	0.01	0.00

**Table 3:** Absorbed doses measured at a point on the surface of the eye without a shield.

POINT NO.	D_1_ [Gy]	D_2_ [Gy]	D_3_ [Gy]	D_4_ [Gy]	D_5_ [Gy]	D_6_ [Gy]	D_7_ [Gy]	D_8_ [Gy]	D_9_ [Gy]	D_10_ [Gy]
1	0.73	1.51	3.04	1.10	1.89	1.86	0.83	0.25	0.31	0.17
2	0.66	1.56	2.14	1.10	1.99	2.22	0.91	0.26	0.39	0.26
3	0.62	1.40	3.01	1.18	1.93	2.28	0.98	0.28	0.49	0.26
4	0.72	1.98	2.67	1.34	1.38	2.08	1.36	0.47	0.34	0.17
5	0.52	1.40	2.56	1.45	1.81	2.28	1.02	0.48	0.37	0.22
6	0.56	1.73	1.74	1.47	1.48	3.10	1.02	0.44	0.44	0.24
7	0.43	1.96	1.94	1.32	1.34	2.88	1.18	0.44	0.40	0.20
8	0.77	1.66	2.11	0.82	1.62	1.76	1.01	0.43	0.44	0.18
9	0.44	1.44	1.77	1.26	1.46	2.14	1.34	0.28	0.42	0.21
10	0.70	1.64	2.28	0.82	2.26	2.47	1.39	0.41	0.45	0.28
**MEAN**	0.62	1.63	2.32	1.19	1.72	2.31	1.11	0.38	0.41	0.22
**STD DEV.**	0.11	0.20	0.45	0.22	0.29	0.40	0.19	0.09	0.05	0.04

**Table 4:** Dose values measured and calculated at a point on the eyelid surface.

	Without Lead Shielding				With Lead Shielding		
PLAN NO.	D_film_ [Gy]	D_TG43_ [Gy]	D_TG186_ [Gy]	D_TG186H_ [Gy]	D_film_ [Gy]	D_TG186_ [Gy]	D_TG186H_ [Gy]
1	0.62	0.76	0.70	0.71	0.57	0.77	0.72
2	1.63	1.91	1.81	1.82	0.40	0.48	0.44
3	2.32	2.88	2.81	2.81	0.75	0.89	0.83
4	1.19	1.46	1.35	1.37	0.10	0.13	0.10
5	1.72	2.12	2.06	2.05	0.22	0.38	0.24
6	2.31	2.80	2.74	2.74	0.18	0.36	0.20
7	1.11	1.28	1.25	1.24	0.09	0.17	0.10
8	0.38	0.46	0.42	0.42	0.08	0.16	0.08
9	0.41	0.55	0.49	0.50	0.06	0.10	0.07
10	0.22	0.31	0.27	0.26	0.04	0.05	0.04

**Table 5:** Doses measured at a point in the lens of the eye—with a shield.

POINT NO.	D_1_ [Gy]	D_2_ [Gy]	D_3_ [Gy]	D_4_ [Gy]	D_5_ [Gy]	D_6_ [Gy]	D_7_ [Gy]	D_8_ [Gy]	D_9_ [Gy]	D_10_ [Gy]
1	0.84	1.41	1.17	0.56	0.44	0.43	0.28	0.14	0.12	0.16
2	0.48	1.08	0.82	0.48	0.41	0.76	0.25	0.15	0.16	0.14
3	0.47	1.34	0.75	0.56	0.32	0.62	0.37	0.13	0.14	0.16
4	0.60	0.98	0.99	0.46	0.48	0.77	0.35	0.14	0.10	0.16
5	0.60	0.96	1.06	0.46	0.53	0.74	0.38	0.09	0.13	0.10
6	0.47	1.12	0.98	0.69	0.34	0.70	0.30	0.10	0.10	0.11
7	0.83	1.12	0.91	0.41	0.33	0.81	0.39	0.11	0.17	0.17
8	0.83	1.04	0.71	0.56	0.56	0.79	0.33	0.12	0.16	0.15
9	0.50	1.41	0.70	0.61	0.34	0.57	0.23	0.10	0.16	0.17
10	0.73	1.31	1.16	0.54	0.43	0.67	0.22	0.08	0.10	0.16
**MEAN**	0.64	1.18	0.93	0.53	0.42	0.69	0.31	0.12	0.13	0.15
**STD DEV.**	0.15	0.17	0.17	0.08	0.08	0.11	0.06	0.02	0.03	0.02

**Table 6:** Doses measured at a point in the lens of the eye without a lead shield.

POINT NO.	D_1_ [Gy]	D_2_ [Gy]	D_3_ [Gy]	D_4_ [Gy]	D_5_ [Gy]	D_6_ [Gy]	D_7_ [Gy]	D_8_ [Gy]	D_9_ [Gy]	D_10_ [Gy]
1	0.86	1.62	1.99	1.88	1.84	3.55	1.69	0.36	0.49	0.28
2	0.63	1.35	1.79	1.59	1.80	2.53	1.49	0.36	0.52	0.20
3	0.66	1.91	2.46	1.36	2.19	3.32	1.25	0.49	0.37	0.35
4	0.62	2.00	2.07	1.39	1.95	2.07	1.29	0.26	0.39	0.34
5	0.86	1.69	2.07	1.43	1.52	2.53	1.27	0.44	0.59	0.30
6	0.75	1.84	1.81	1.30	1.60	2.86	1.78	0.42	0.57	0.30
7	0.83	1.42	2.46	1.39	1.64	3.78	1.17	0.34	0.42	0.25
8	0.61	1.42	1.68	1.68	1.85	2.07	1.56	0.26	0.56	0.24
9	0.46	2.15	2.37	2.01	1.91	2.14	1.43	0.49	0.54	0.30
10	0.61	1.47	1.90	1.46	1.22	3.26	1.38	0.33	0.46	0.27
**MEAN**	0.69	1.69	2.06	1.55	1.75	2.81	1.43	0.38	0.49	0.28
**STD DEV.**	0.13	0.26	0.27	0.23	0.25	0.60	0.19	0.08	0.07	0.04

**Table 7:** Dose values measured and calculated at a point in the lens of the eye.

	Without Lead Shielding				With Lead Shielding		
PLAN NO.	D_film_ [Gy]	D_TG43_ [Gy]	D_TG186_ [Gy]	D_TG186H_ [Gy]	D_film_ [Gy]	D_TG186_ [Gy]	D_TG186H_ [Gy]
1	0.69	0.81	0.75	0.76	0.64	0.75	0.76
2	1.69	2.31	2.23	2.22	1.18	1.23	1.22
3	2.06	2.22	2.12	2.16	0.93	1.05	1.06
4	1.55	1.86	1.78	1.81	0.53	0.62	0.60
5	1.75	2.03	1.96	1.97	0.42	0.43	0.49
6	2.81	3.34	3.25	3.29	0.69	0.75	0.71
7	1.43	1.66	1.60	1.60	0.31	0.28	0.34
8	0.38	0.49	0.43	0.43	0.12	0.12	0.12
9	0.49	0.55	0.51	0.51	0.13	0.14	0.14
10	0.28	0.34	0.30	0.30	0.15	0.15	0.14

## Data Availability

Not applicable.
